# Possible Volumetric Changes in the Hippocampus and Olfactory Bulbs After COVID-19: A Stereological MRI Study

**DOI:** 10.3390/v18080902

**Published:** 2026-08-16

**Authors:** Gamze Altun, Aynur Atilla, Kerim Aslan, Kıymet Kübra Tüfekci, Barış Genç, Arife Ahsen Kaplan, Özgür Kemal, Murat Terzi, Hüseyin Akan, Süleyman Kaplan

**Affiliations:** 1 Department of Histology and Embryology, Faculty of Medicine, Ondokuz Mayıs University, 55139 Samsun, Türkiye; skaplanomu@yahoo.com; 2 Department of Infection Diseases, Faculty of Medicine, Ondokuz Mayıs University, 55139 Samsun, Türkiye; aynura@omu.edu.tr; 3 Department of Radiology, Faculty of Medicine, Ondokuz Mayıs University, 55139 Samsun, Türkiye; kerim.aslan@omu.edu.tr (K.A.); baris.genc@omu.edu.tr (B.G.); akanhus@yahoo.com (H.A.); 4 Department of Histology and Embryology, Faculty of Medicine, Kastamonu University, 37150 Kastamonu, Türkiye; kkyurt@kastamonu.edu.tr; 5 Department of Histology and Embryology, Faculty of Medicine, Bolu Abant İzzet Baysal University, 14030 Bolu, Türkiye; ahsenkaplan@gmail.com; 6 Department of Otolaryngology, Faculty of Medicine, Ondokuz Mayıs University, 55139 Samsun, Türkiye; ozgur.kemal@omu.edu.tr; 7 Department of Neurology, Faculty of Medicine, Ondokuz Mayıs University, 55139 Samsun, Türkiye; mterzi@omu.edu.tr

**Keywords:** COVID-19, hippocampus, magnetic resonance imaging, olfactory bulb, Cavalieri principle, stereology

## Abstract

COVID-19 affects the olfactory system and the hippocampus, leading to severe olfactory and cognitive impairments. This study aimed to quantify volumetric changes in the olfactory bulbs and hippocampus in patients with COVID-19, and to investigate these differences in relation to loss of smell and taste. This stereological MRI study included 60 COVID-19 (+) participants and 54 non-COVID-19 participants. Using OsiriX software, the volumes of the olfactory bulb and hippocampus were manually measured from 3T MRI images. Compared with controls, participants with loss of smell and taste had smaller olfactory bulb volumes. This decrease was especially noticeable in the left olfactory bulb of female patients. Furthermore, the right hippocampal volume in male patients with loss of smell and taste was significantly decreased. These findings suggest that atrophy of the olfactory bulb and hippocampus may be specific to certain regions and sexes following SARS-CoV-2 infection. Stereological MRI assessments can effectively identify these changes. Prospective longitudinal studies are needed to determine the clinical significance of these changes and their impact on patient management.

## 1. Introduction

Coronavirus disease 2019 (COVID-19) continues to have a wide-ranging impact on social and human health. The primary cause of COVID-19 is SARS-CoV-2, a highly pathogenic virus that causes pneumonia-like symptoms, including fever and cough [1,2]. The results underscore the importance of long-term neuroimaging surveillance for COVID-19 patients exhibiting olfactory symptoms. According to reports, between 10 and 20 percent of COVID-19 patients have chronic symptoms that last longer than a bulb, including headaches, shortness of breath, and reduced neurocognitive performance [3]. In 2020, the term “long COVID”—also known as “post-COVID-19”—was introduced to describe symptoms that persist after the initial phase [4]. Atypical appearances, drug side effects, or psychosocial aspects of the COVID-19 course can make diagnosing post-COVID syndrome challenging [5]. Current sources indicate a risk of COVID-19 resurging in 2025, but the extent of this risk remains unclear [6]. There is still considerable concern regarding the health effects of COVID-19. Morphological changes are observed throughout the olfactory plexus, including the olfactory bulb, in patients with COVID-19, and loss of taste and smell is significant [7].

Additionally, it has been emphasized that verbal areas are affected at the cognitive level in the short- and long-term after COVID-19 [8]. In the literature, changes in brain volume after COVID-19 are contradictory. Some studies report an increase in volume, while others report a decrease [9]. In this context, our study, by considering the stereological approaches we used and gender-specific measurements, may offer a new perspective on the knowledge by evaluating hippocampus and olfactory bulb volumes together. In recent years, the belief that SARS-CoV-2 directly affects the brain and olfactory bulb has been incorrect. COVID-19-related neurological changes may be associated with mechanisms of neuroinflammation [10]. Current evidence suggests that olfactory loss is mediated by peripheral nervous system mechanisms rather than by neuroinvasion of SARS-CoV-2 [10,11,12]. The most likely mechanism for the volume change may be atrophy resulting from disuse of the olfactory and limbic areas. Even the elimination of olfactory stimuli alone can lead to degeneration of the olfactory bulb. In this context, olfactory deficiency can re-modify the brain’s structure and functional relationships. At a more secondary level, the limbic system, including the hippocampus, is also affected [13,14,15].

This study investigates potential brain damage resulting from COVID-19 infection by examining morphological alterations in patients with the disease. The research examines gender differences and investigates the neuroanatomical association between the olfactory bulb and hippocampus in this neuropathological problem. This morphoquantitative investigation of sexual dimorphism provides new insights and may significantly enhance the current research.

## 2. Materials and Methods

### 2.1. Group Design and Data Collection

All procedures received approval from the Clinical Research Ethics Committee of Ondokuz Mayıs University (approval date: 8 October 2020, decision number: 2020/545). The research was conducted in accordance with the ethical standards of the Declaration of Helsinki. This prospective study involved contacting individuals diagnosed with COVID-19 between 1 March 2021 and 8 September 2022, either in person or by telephone. The study details were explained to each participant, and informed consent was obtained. MIA Technology Hospital Information Management System Software (MIA Technology, Ankara/Türkiye) was used to collect the contact information of male and non-pregnant female patients aged 18 to 65 who visited the University Hospital COVID-19 Unit. The power analysis to determine the number of individuals per group was performed using G*Power 3.1. Within the scope of the analysis, the “ANOVA: Fixed effects, omnibus, one-way” test was selected under the “F tests” family. The number of individuals in each group was determined using the G*power analysis program. The significance level (α error probability) of the study is 0.05, and the effect size (f) value of the study is 0.5738. Considering these values, there are 8 individuals per group. As a result of the analysis, the model’s actual power was found to be 0.8348.

No randomization technique was used for group assignment; participants were chosen voluntarily. Participants were contacted by phone, provided with general project details, and informed of the necessary procedures. MRI scans were performed one to three months after RT-PCR confirmed the COVID-19 infection in the patient cohorts. The patient’s availability and the MRI unit’s capacity at the Ondokuz Mayıs University, Faculty of Medicine, Radiology Department were taken into consideration when choosing the date. Consenting individuals were scheduled for MRI appointments, and brain MRI scans were performed in the Radiology Department using a 3T MRI scanner with a 32-channel head coil (Philips Ingenia; Philips Healthcare, The Netherlands). About fifty to sixty minutes were needed for each MRI scan. Patients in the control group had MRIs for mild conditions like headaches, migraines, or moderate trauma and showed no abnormal results. Stereological techniques were used to analyze the control MRI images obtained between 2020 and 2022. Participants were excluded if they were not between the ages of 18 and 65, had a history of surgery, had cancer, had hemiplegia, had intracranial tumors, had neurological conditions (such as multiple sclerosis, amyotrophic lateral sclerosis, dementia, or Parkinson’s disease), or were pregnant or nursing. Table 1 provides specifics on the inclusion and exclusion criteria.

Loss of smell and taste was assessed based on patient self-reports within the scope of clinical evaluation conducted at the time of RT-PCR-confirmed COVID-19 diagnosis. The duration of chemosensory dysfunction reported by COVID-positive patients at the time of examination ranged from 1 to 3 months. Participants reporting partial or complete loss of smell and taste were included in the loss of smell and taste group. The final analysis excluded patients who met the study’s requirements but did not have MRI scans or who withdrew their consent after learning they were pregnant and nursing. Table 2 displays the mean age of each group and the distribution of patients who had MRIs. People with the loss of taste and smell are denoted by the acronym A-A (Anosmia–Ageusia, smell and taste) in Table 2.

### 2.2. Magnetic Resonance Imaging Protocol

The imaging protocol for all patients included sT1W_3D_TFE, 3D_Brain_VIEW_T2, and 3D_Brain_VIEW_FLAIR sequences. Imaging parameters for the sT1W_3D_TFE and 3D Brain VIEW T2 sequences used in stereological analysis are detailed in Table 3 and Table 4. In Table 3, Bandwidth is indicated by “BW”, Field of View is indicated by “FOV”, Echo time is indicated by “TE”, Repetition time is indicated by “TR”, Radio frequency is indicated by “RF”, and Water-fat shift is indicated by “WFS”. Additionally, In Table 4, Acquisition with “ACQ”, Anterior–posterior with “AP”, Bandwidth with “BW”, Foot–head with “FH”, Field of view with “FOV”, Peripheral nerve stimulation with “PNS”, Right–left with “RL”, Specific absorption rate with “SAR”, Specific energy dose with “SED”, Echo time with “TE”, Turbo spin-echo with “TSE”, Effective echo time is specified with “TEeff”, Equivalent echo time with “TEequiv”, Repetition time with “TR”, Volumetric pixel with “Voxel”, and Water-fat shift (per pixel) with “Wfs (pix)”.

### 2.3. Stereological Analyses

**Figure 1:**
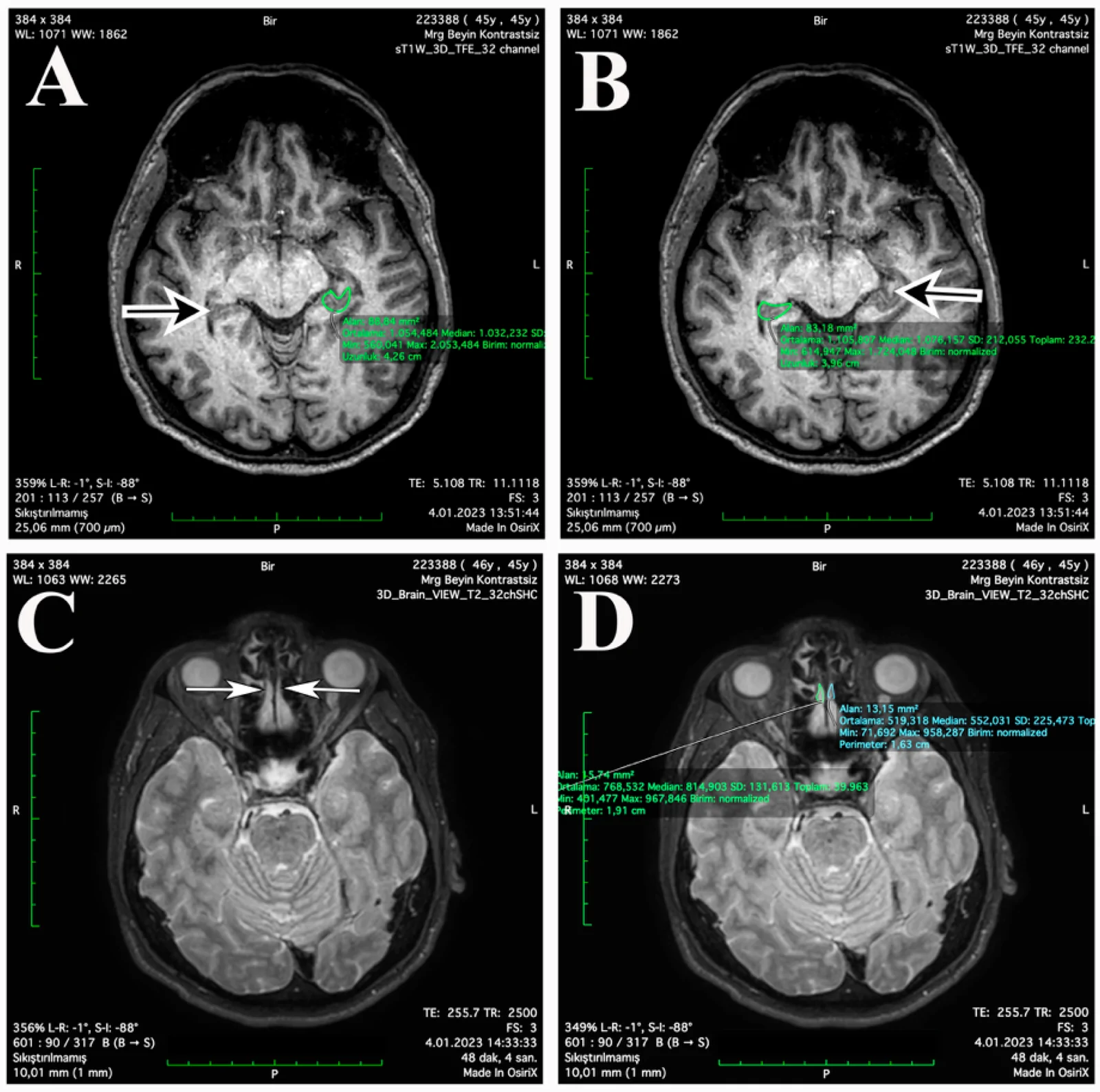
The planimetric analysis of the olfactory bulb and hippocampus regions in the MR images obtained from the sT1W_3D_TFE and 3D_Brain_VIEW_T2 sequences is presented. (**A**,**B**) Measurements in Osirix Lite of hippocampal and (**C**,**D**) olfactory bulb regions. Black arrow: hippocampus; white arrow: olfactory bulb.

Hippocampal formation and olfactory bulb volumes were obtained from serial coronal 3D MR images using the OsiriX Lite (version 13.0.2, Geneva, Switzerland) program and measured. The hippocampus was evaluated in sequential coronal sections, and anatomical boundaries were drawn manually. Segmentation included the cornu ammonis (CA) 1, CA2, CA3, CA4, and subiculum portions that make up the hippocampal formation. The olfactory bulb was evaluated using sequential coronal T2-weighted images. Segmentation of the olfactory bulb was performed using sequential coronal T2-weighted images, with the first section separating it from the olfactory tract being considered. Volume measurements were performed until the last section, where the olfactory bulb could no longer be seen. To perform the volumetric analyses, the MRI images from the control and patient groups were loaded into OsiriX Lite (version 13.0.2, Geneva, Switzerland). The olfactory bulb and hippocampal volumes were segmented manually on coronal T2-weighted images using OsiriX Lite DICOM Viewer (version 13.0.2, Switzerland), a DICOM viewer and analysis tool. All volumetric measurements were performed manually by a researcher with adequate training and experience in neuroanatomy and stereological analysis at the MRI level. Stereological analysis was performed blindly. Analyses were performed blind as to cohort type by two observers. On 3D-T1W (TE: 5.108 ms, TR: 11.11 ms, matrix: 384 × 384) and 3D T2W (TE: 255.7 ms, TR: 2500 ms, matrix: 384 × 384) MR images, the surface areas of the olfactory bulb and hippocampal regions were measured planimetrically. In total, 23 out of 257 slices in the 3D-T1W sequence had hippocampus regions (slice thickness 0.7 mm, gap 0.7 mm), whereas 4 out of 257 slices in the 3D-T2W sequence had olfactory bulb regions (slice thickness 1 mm, gap 0.5 mm). Slice thickness and slice gap were added together to calculate volume (Figure 1A–D).

### 2.4. Statistical Analyses

Data analysis was done with GraphPad Prism (version 10.2). The Kolmogorov–Smirnov tests were used to assess the normality of the distributions. A significance level of 0.05 was applied; data were deemed non-normally distributed if *p* < 0.05. For multiple comparisons, the Kruskal–Wallis H test and Dunn’s post hoc test were used; significance was defined as *p* < 0.05. After the Kruskal–Wallis test showed statistical significance, Dunn’s multiple comparisons test and adjusted *p*-values were used. In addition, multiple linear regression analyses were performed to control for potential confounding effects of age and gender on the hippocampus and olfactory bulb. Age and gender-adjusted group effects were evaluated for each volume measurement. Regression coefficients (β), 95% confidence intervals, and *p*-values were reported. Multicollinearity was assessed using Variance Inflation Factor (VIF) values. Statistical significance was accepted at *p* < 0.05.

## 3. Results

To compare stereological data, “mean ± standard deviation (SD)” values were employed. Due to the sample size, both the skewness–kurtosis coefficients and the Kolmogorov–Smirnov test were employed for normality testing. The results of the Kolmogorov–Smirnov test indicated that the measurement levels were not normally distributed (*p* < 0.05). If the skewness and kurtosis values are between −1.50 and +1.50, a normal distribution can be presumed [16]. These criteria indicated that the measurement levels were not normally distributed, suggesting that nonparametric methods are suitable for testing differences, correlations, and comparisons.

### 3.1. Age- and Gender-Adjusted Analyses

Multiple linear regression analyses were performed to evaluate whether differences in hippocampal and olfactory bulb volumes persisted independent of age and gender. No significant independent effect of age and gender was found on the left, right, and total hippocampal volumes (all *p* > 0.05). In contrast, after age and gender were included in the model, the overall effect of the study group on the left, right, and total hippocampal volumes remained significant (all *p* < 0.0001). Compared with the control group without a COVID-19 diagnosis, left hippocampal volume was significantly lower in both groups of patients diagnosed with COVID-19, with (β = −1929, *p* < 0.0001) and without (β = −1933, *p* < 0.0001) loss of smell and taste. Similarly, right hippocampal volume was significantly smaller in both COVID-19 groups than in the control group (β = −1887 and −1799, respectively; *p* < 0.0001 for both). Regarding total hippocampal volume, a significant decrease was also observed in both the group of patients diagnosed with COVID-19 who did not have loss of smell and taste (β = −3816, *p* < 0.0001) and those who did have loss of smell and taste (β = −3731, *p* < 0.0001) compared to the control group without a COVID-19 diagnosis. Regression models explained 65.21%, 64.78%, and 66.42% of the variance in the left, right, and total hippocampal volumes, respectively. Similarly, age and sex had no significant independent effects on the left, right, or total olfactory bulb volumes (all *p* > 0.05). In contrast, the overall effect of the study group was significant for the left (*p* < 0.0001), right (*p* = 0.0026), and total olfactory bulb volumes (*p* = 0.0002). Compared with the control group without a COVID-19 diagnosis, the patient group with COVID-19 and loss of smell and taste had significantly lower left (β = −12.77, *p* < 0.0001), right (β = −10.14, *p* = 0.0006), and total olfactory bulb volumes (β = −22.92, *p* < 0.0001). In contrast, no significant difference was found between the COVID-19 diagnosed patient group without loss of smell and taste and the control group without a COVID-19 diagnosis in terms of left (β = −5.177, *p* = 0.0812), right (β = −3.554, *p* = 0.2472), and total olfactory bulb volumes (β = −8.736, *p* = 0.1309).

### 3.2. Volumes of the Right and Left Olfactory Bulb

**Figure 2:**
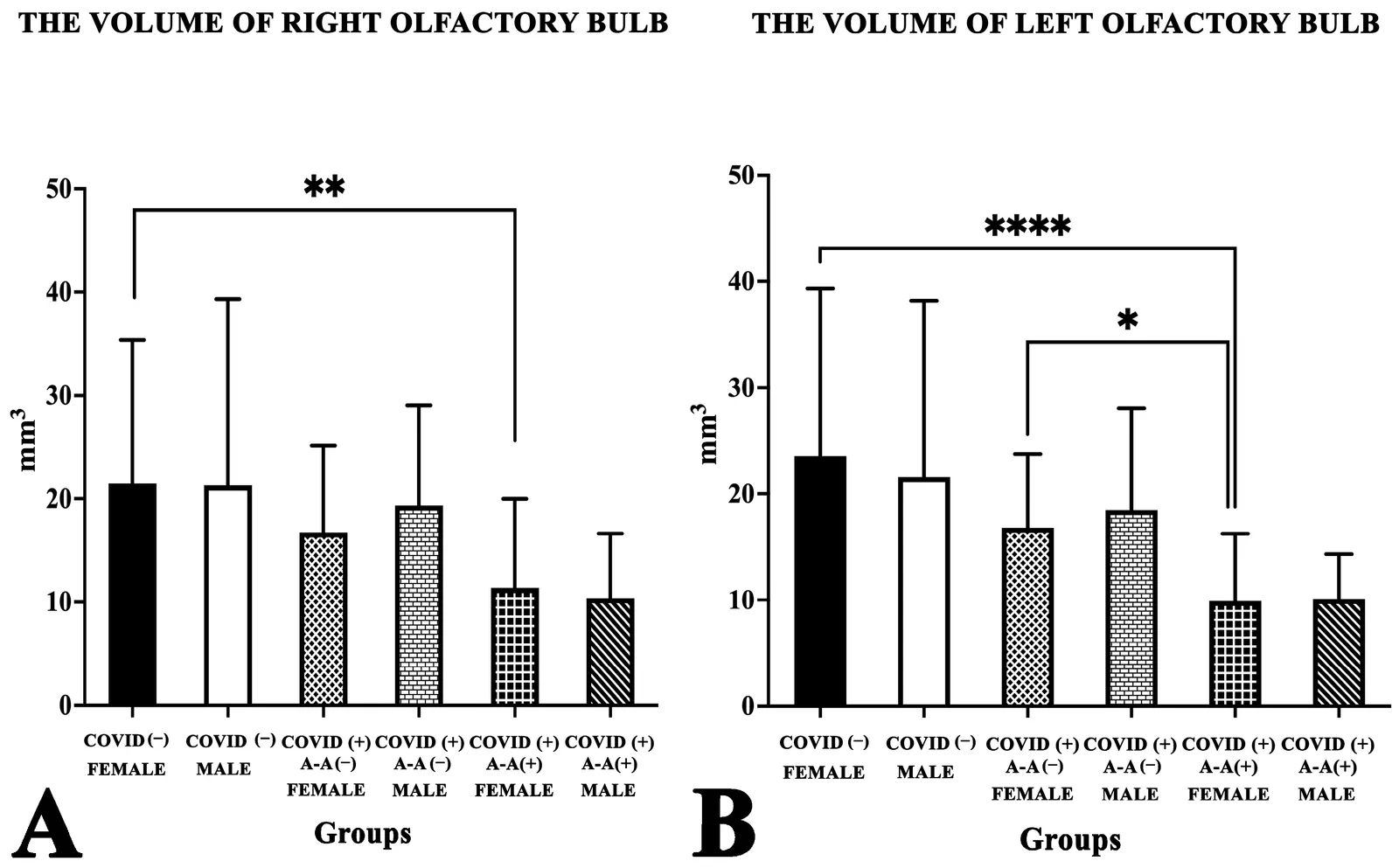
(**A**,**B**) The mean ± SD values of the groups’ right and left olfactory bulb volumes (mm^3^) are shown in the graph. Statistical differences between groups are indicated with “*, **, ****.” *: *p* < 0.05, **: *p* < 0.01, ****: *p* < 0.0001. A-A (−): No loss of taste and smell; A-A (+): With loss of taste and smell.

The comparison of right olfactory bulb volumes between groups yielded a test statistic (H) of 22.305 and a *p*-value of 0.001, indicating statistical significance. A significant difference was seen between the groups (*p* < 0.05). The volume of the right olfactory bulb in the COVID (+) Female (A-A (+)) group was statistically smaller than that in the COVID (−) Female group (*p* = 0.0024). For left olfactory bulb volume, the test statistic (H) is 32.445 with a *p*-value of 0.001, indicating statistical significance. A notable discrepancy was seen between the groups (*p* < 0.05). A statistically significant reduction in the left olfactory bulb volume was seen in the COVID (+) Female (A-A (+) group compared to the COVID (+) Female (A-A (−) group (*p* = 0.04). It was also noted that the left olfactory bulb volume for the COVID (+) Female (A-A (+) group decreased compared to the Female control group (*p* < 0.0001) (Table 5, Figure 2A,B).

### 3.3. Volumes of the Right and Left Hippocampus

**Figure 3:**
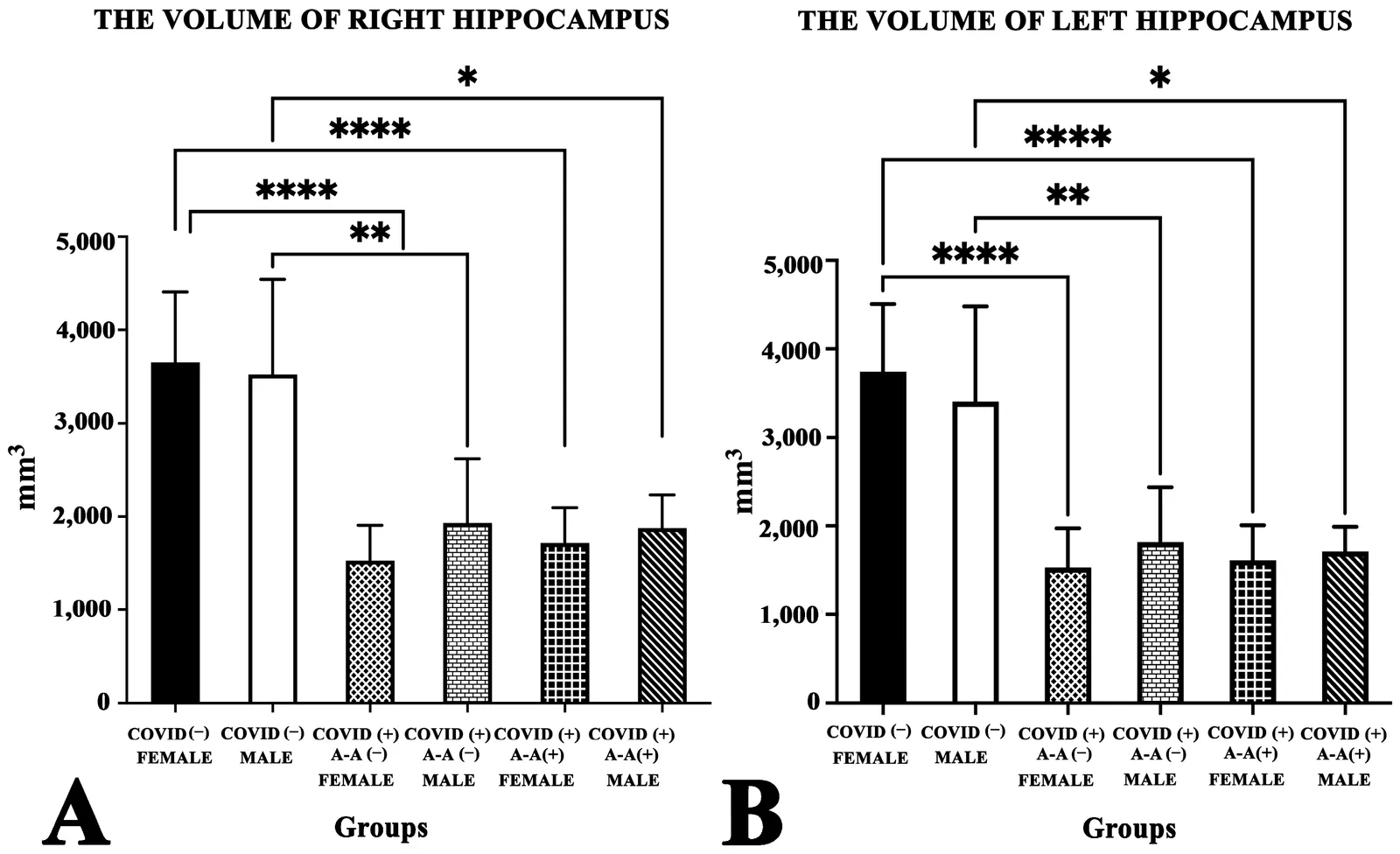
(**A**,**B**) The mean ± SD values of the right and left hippocampal volumes (mm^3^) of the groups are shown in the graph. Statistical differences between groups are indicated with “*, **, ****.” *: *p* < 0.05, **: *p* < 0.01, ****: *p* < 0.0001. A-A (−): No loss of taste and smell; A-A (+): With loss of taste and smell.

A statistically significant difference was observed between the groups in right hippocampal volumes (H = 76.732, *p* < 0.05). The right hippocampal volumes of the COVID (−) female control group were significantly greater than those of COVID (+) female patients (A-A (+), A-A (−)) (*p* < 0.0001). The right hippocampal volumes of the COVID (−) male (control male) group were significantly greater than those of COVID (+) male patients (A-A (+), A-A (−)) (respectively, *p* = 0.0143, *p* = 0.0022). A significant difference in the left hippocampal volume was observed across groups (H = 71.374, *p* < 0.05). The left hippocampal volumes of the COVID (−) Female (Control female) group were substantially greater than those of COVID (+) female patients (A-A (+), A-A (−)) (*p* < 0.0001). The left hippocampal volumes of the COVID (−) Male (Control Male) group were significantly greater than those of COVID (+) male patients (A-A (+), A-A (−)) (respectively, *p* = 0.0178, *p* = 0.0081) (Table 6, Figure 3A,B).

### 3.4. Total Olfactory Bulb and Total Hippocampus Volumes

Analysis regarding total olfactory bulb volume revealed a significant difference between groups (H = 31.983, *p* < 0.05). There is no significant difference between the total olfactory bulb volumes in the COVID (+) Male (A-A (+) and the Male control group (*p* = 0.05). The total olfactory bulb volume in the COVID (+) Female (A-A (+) group was significantly smaller than that of the COVID (−) Female control group (*p* < 0.0001).

**Figure 4:**
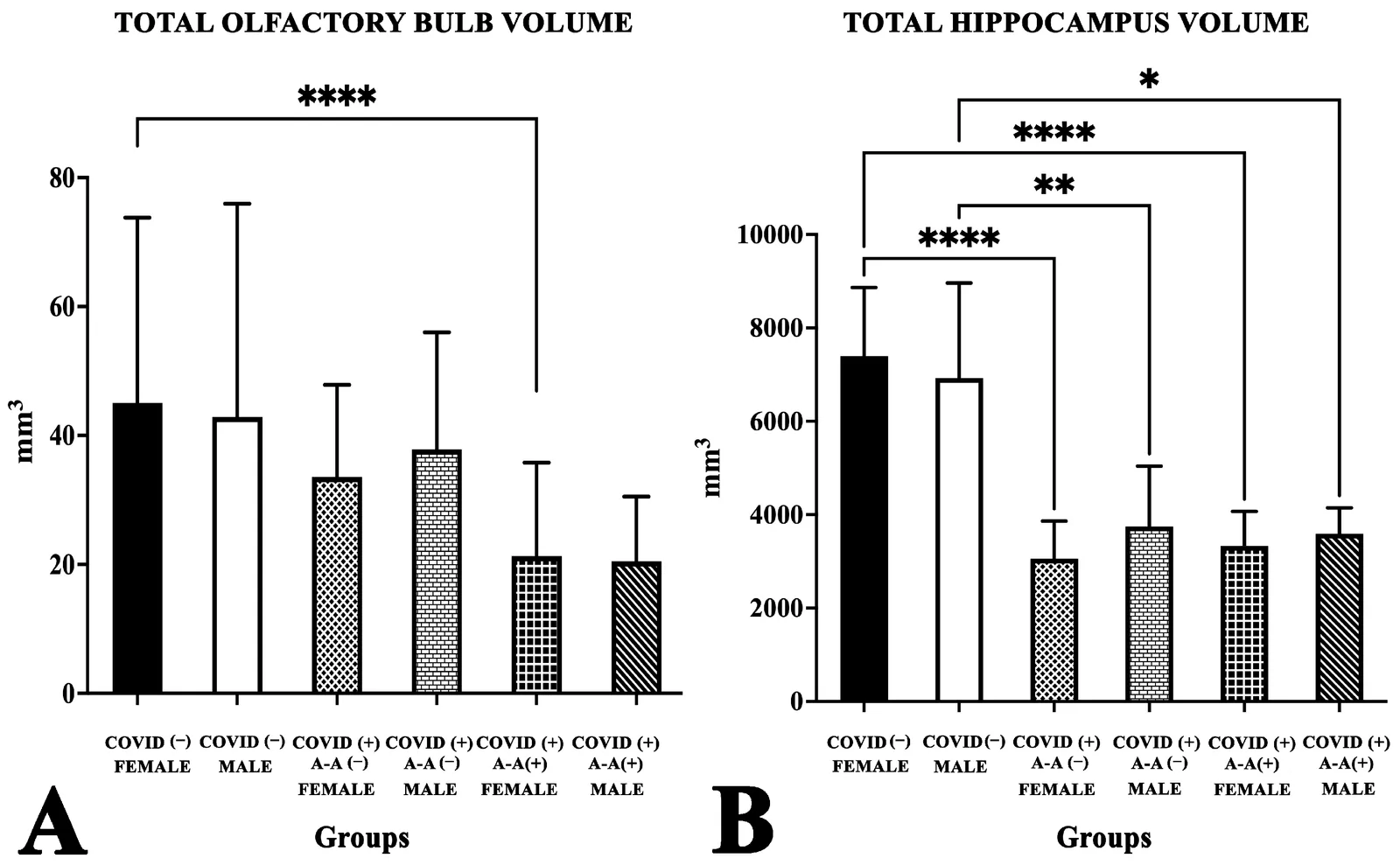
(**A**,**B**) The mean ± SD values of the groups’ total olfactory bulb volume and total hippocampal volume (mm^3^) are shown in the graph. Statistical differences between groups are indicated with “*, **, ****.” *: *p* < 0.05, **: *p* < 0.01, ****: *p* < 0.0001. A-A (−): No loss of taste and smell; A-A (+): With loss of taste and smell.

A significant difference in total hippocampal volume was observed between the groups (H = 74.995, *p* < 0.05). The total hippocampal volumes in the COVID (−) female group were significantly greater compared to those in COVID (+) female patients (A-A (+) and A-A (−) (*p* < 0.0001). The total hippocampal volumes in the COVID (−) male group were higher than those in COVID (+) male patients (A-A (+), A-A (−)), with statistical significance (respectively, *p* = 0.019 and *p* = 0.0037) (Table 5 and Table 6, Figure 4A,B).

## 4. Discussion

The prolonged cytokine storm triggered by SARS-CoV-2 has intensified the hyperinflammatory response, contributing to cardiovascular and neurovascular pathologies and accelerating neurodegeneration [17]. Following COVID-19, a significant loss of cognitive function, usually expressed as “brain fog,” persists long-term [18]. Cognitive decline in brain regions connected to memory and perception appears to affect the same regions targeted by Alzheimer’s disease [17].

COVID-19’s long-term effects, referred to as “long-COVID-19” or “post-COVID-19” symptoms, include learning and memory problems, as well as neuropsychiatric challenges such as post-traumatic stress disorder. Cognitive problems in long-term COVID-19 patients, particularly due to neuroinflammation, can emerge with time [18]. The hippocampus’s susceptibility to neurological and behavioral diseases makes it particularly vulnerable to COVID-19-related damage. Research suggests neurodegenerative changes after infection that resemble Alzheimer’s disease pathology [19,20].

Inhibited neurogenesis has been linked to microglial activation in patients with long COVID-19. Following SARS-CoV-2 infection, microglia—which maintain synapses and support neurons in the central nervous system—become abnormally reactive, disrupting CNS homeostasis and potentially impairing cognitive function by suppressing neurogenesis [21]. Hippocampal neurons are harmed by oxidative stress caused by neuroinflammation, which results from SARS-CoV-2 activation of microglia and astrocytes [22,23,24,25].

With the COVID-19 pandemic, significant differences have been observed between genders in endocrine balance and immune responses [26,27]. Women demonstrate a higher susceptibility to chronic immune irregularities and autoimmune diseases, while men show more significant homeostatic disturbances during acute viral infections. The impact of COVID-19 on brain homeostasis has led to a higher prevalence of post-COVID-19 symptoms among women [28]. Post-infection memory impairments have been linked to changes in gray matter volume and metabolic processes [29].

Postmortem studies indicate that hippocampal abnormalities associated with COVID-19 are likely linked to neuroinflammation, as evidenced by changes in neuronal and glial morphology [30,31]. Our study revealed a bilateral decrease in hippocampal volume in both female and male patients, regardless of smell loss, compared with controls. Qin et al. (2021) found cortical thinning in the hippocampus region three months after SARS-CoV-2 infection [24]. A study reported decreased gray matter volumes in regions associated with cognitive and olfactory impairments resulting from COVID-19, including the hippocampus, amygdala, lingual gyrus, and other essential brain regions [32].

Bilateral effects in the olfactory bulb may not occur in male patients. Hippocampal volume reduction was observed in both male and female post-COVID (+) patients. Loss of smell and taste, frequently reported in COVID-19, may persist for weeks and significantly impact quality of life, even in the absence of other symptoms such as cough, fever, and fatigue [33,34]. MRI is crucial for assessing olfactory loss and investigating associated anatomical structures [7]. Our study found bilateral reductions in olfactory bulb volume in female patients with olfactory and gustatory dysfunction. In males, the left olfactory bulb volume decreased, while no change was observed in the right. The absence of a bilateral effect in male patients may be more closely related to sample size than to a simple gender difference.

Stereological methodologies provide evidence for the impact of COVID-19 on memory and olfaction; however, several limitations hinder the interpretation of the findings. A primary weakness of our study is the small sample size of the male cohort. This may pertain to the diminished adherence to MRI sessions among male patients with post-COVID-19 symptoms, including loss of smell and taste. Our research concentrated mostly on morphological metrics. The lack of clinical cognitive evaluations constitutes another limitation of our investigation. Future studies will investigate the correlation between clinical manifestations and structural alterations in patients post-COVID-19. A further limitation of the study is the lack of documentation of participants’ educational attainment. Recent studies indicate that this relationship may not be significant. Nyberg et al. (2021) reported that individuals’ educational level does not influence the rate of change in cortical and hippocampal structures [35]. Similarly, Lövdén et al. (2023) found that volumetric changes in the hippocampus and episodic memory are not associated with education [36]. The primary inquiry of our research is whether the reduction in olfactory bulb volume observed in COVID-19 patients correlates with the loss of smell and taste, and how the molecular mechanisms underlying this impact on hippocampal function operate. Another limitation of the study is that objective psychophysical tests for olfactory and gustatory disorders could not be applied. Although reductions in hippocampal volume are associated with cognitive impairments, the limitation of our study—the absence of neurocognitive tests—prevents us from drawing a direct conclusion regarding cognitive dysfunction. Further studies are planned using tests and diagnostic criteria that demonstrate neurocognitive activity. In this context, our study can be considered a preliminary morphometric assessment of the olfactory bulb and hippocampus in terms of volumetric measures.

Further multiple regression analysis, aimed at determining whether the observed volume changes were not influenced by age and gender, demonstrated that neither age nor gender had a significant independent effect on the volumes of the hippocampi and olfactory bulbs examined. The fact that the group difference remained the same indicates that the changes in volume cannot be attributed solely to these demographic factors. In particular, the maintenance of the reduction in the volumes of the left and right sides of the olfactory bulb and its total volume in the COVID-19 group with loss of smell and taste, in comparison with the control group of patients with COVID-19 without loss of smell and taste, may indicate that the changes in the olfactory bulb volume are more likely connected to clinical chemosensory dysfunction. At the same time, the decrease in hippocampal volume in both COVID-19 groups compared with controls may suggest that hippocampal changes are not solely associated with loss of smell and taste.

## 5. Conclusions

Gender differences may affect loss of smell and taste, with hormonal factors possibly playing a role in its pathophysiology. Our study found that female patients exhibited a more significant loss of smell and taste, which correlated with decreased olfactory bulb volume. Bilateral reductions in olfactory bulb and hippocampal volumes among COVID-19-positive individuals of both genders may suggest potential cognitive implications. Additional research is needed to elucidate the mechanisms underlying the reduction in olfactory bulb volume in female patients with COVID-19, including potential hormonal effects and gender-specific neuroinflammatory responses.

## Figures and Tables

**Table 1:** Inclusion Criteria.

Male and female patients aged 18–65 years
The control group included patients who underwent MRI for minor reasons (e.g., headache, migraine, minor trauma) and had no pathological findings
Having a COVID-19 (+) diagnosis confirmed by RT-PCR test
COVID-19-positive patients (with or without taste and smell loss) who did not have a diagnosis of cancer, hemiplegia, intracranial tumors, neurological disease, or psychiatric conditions
Female patients who were not pregnant or breastfeeding

**Table 2:** Group design and average age of the groups.

**COVID-19 (+) Patients**	**Female**		**Male**	
	**A-A (+)** **(*n* = 25)**	**A-A (−)** **(*n* = 16)**	**A-A (+)** **(*n* = 9)**	**A-A (−)** **(*n* = 10)**
**Average age**	38.38	40.438	48.80	42.90
**COVID-19 (−) Patients** **(Control group)**	**Female** **(*n* = 25)**		**Male** **(*n* = 29)**	
**Average age**	37.23		45.97	

Participants reporting loss of smell and taste were classified as A-A (+), while those without loss of smell or taste were classified as A-A (−).

**Table 3:** Three-dimensional MR imaging parameters of the sT1W_3D_TFE sequence.

Parameters	
Field strength (T)	3
RF Coil configuration	32-channel-head coil
Scanning Sequence	sT1W_3D_TFE
Orientation	Coronal
TE/TR [ms]	5.18 ms/11.27 ms
Flip angle	8°
FOV [mm^2^]	AP = 256, RL = 240
Number of Slice	214
Slice thickness [mm]	0.7 mm
Acquisition voxel size [mm^3^]	AP = 0.699999988. RL = 0.695652008. FH = 0.699999988
WFS (pix)/BW (Hz)	3.261 → 133.2

**Table 4:** Three-dimensional MR imaging parameters of the 3D_Brain_VIEW_T2.

Parameters	
FOV AP	250
FOV RL	197.916672
FOV FH	175
ACQ voxel size AP, RL, FH	1, 1, 1
Recon voxel size AP, RL, FH	0.651041687, 0.651041687, 0.5
Reconstruction matrix	384
TSE factor	117
TE	Shortest
TR	2500
Actual TR	2500
Actual TR	262
ACQ matrix M × P	252 × 198
Actual slice gap	−0.5
Slice thickness	1 mm
WFS (pix)/BW (Hz)	0.552/787.3
TSEes/shot	3.9/488
TEeff/TEequiv	262/110
Minimum TR	515

**Table 5:** Mean ± SD (mm^3^) values for olfactory bulb volume estimations.

Variable	Groups	Mean ± SD (mm^3^)
Right olfactory bulb volume	COVID (+) Female A-A (+)	11.37 ± 8.62
	COVID (+) Female A-A (−)	16.76 ± 8.39
	COVID (+) Male A-A (+)	10.38 ± 6.24
	COVID (+) Male A-A (−)	19.35 ± 9.66
	COVID (−) Male	21.30 ± 18.01
	COVID (−) Female	21.47 ± 13.88
Left olfactory bulb volume	COVID (+) Female A-A (+)	9.91 ± 6.34
	COVID (+) Female A-A (−)	16.82 ± 6.95
	COVID (+) Male A-A (+)	10.10 ± 4.21
	COVID (+) Male A-A (−)	18.49 ± 9.56
	COVID (−) Male	21.57 ± 16.61
	COVID (−) Female	23.57 ± 15.77
Total olfactory bulb volume	COVID (+) Female A-A (+)	21.28 ± 14.52
	COVID (+) Female A-A (−)	33.58 ± 14.26
	COVID (+) Male A-A (+)	20.48 ± 10.05
	COVID (+) Male A-A (−)	37.84 ± 18.15
	COVID (−) Male	42.87 ± 33.07
	COVID (−) Female	45.04 ± 28.74

A-A (−): No loss of taste and smell; A-A (+): With loss of taste and smell.

**Table 6:** Mean ± SD (mm^3^) values for hippocampal volume estimations.

Variable	Groups	Mean ± SD (mm^3^)
Right hippocampus volume	COVID (+) Female A-A (+)	1717.64 ± 376.58
	COVID (+) Female A-A (−)	1530.30 ± 375.28
	COVID (+) Male A-A (+)	1877.16 ± 353.74
	COVID (+) Male A-A (−)	1930.12 ± 691.43
	COVID (−) Male	3522 ± 1021
	COVID (−) Female	3652.48 ± 758.88
Left hippocampus volume	COVID (+) Female A-A (+)	1613.97 ± 393.40
	COVID (+) Female A-A (−)	1532.44 ± 440.21
	COVID (+) Male A-A (+)	1713.20 ± 277.55
	COVID (+) Male A-A (−)	1817.80 ± 617.82
	COVID (−) Male	3743 ± 766.8
	COVID (−) Female	3742.74 ± 766.77
Total hippocampal volume	COVID (+) Female A-A (+)	3331.61 ± 738.12
	COVID (+) Female A-A (−)	3062.74 ± 800.84
	COVID (+) Male A-A (+)	3590.35 ± 556.51
	COVID (+) Male A-A (−)	3747.91 ± 1292.93
	COVID (−) Male	6928 ± 2034
	COVID (−) Female	7395.22 ± 1471.24

A-A (−): No loss of taste and smell; A-A (+): With loss of taste and smell.

## Data Availability

The data presented in this study are available on request from the corresponding author.

## Abbreviations

The following abbreviations are used in this manuscript:

BW	Bandwidth
COVID-19	Coronavirus Disease 2019
FOV	Field of View
TE	Echo time
TR	Repetition time
RF	Radio frequency
WFS	Water-fat shift
ACQ	Acquisition
AP	Anterior–posterior
RL	Right-left
SAR	Specific absorption rate
TSE	Turbo spin-echo
FH	Foot-head
SED	Specific energy dose
PNS	Peripheral nerve stimulation
Voxel	Volumetric pixel
